# Hyperkeratotic hand eczema: Eczema or not?

**DOI:** 10.1111/cod.13572

**Published:** 2020-06-01

**Authors:** Klaziena Politiek, Laura Loman, Hendri H. Pas, Gilles F.H. Diercks, Henny H. Lemmink, Sabrina Z. Jan, Peter C. van den Akker, Maria C. Bolling, Marie L.A. Schuttelaar

**Affiliations:** ^1^ Department of Dermatology, University Medical Center Groningen University of Groningen Groningen The Netherlands; ^2^ Department of Pathology, University Medical Center Groningen University of Groningen Groningen The Netherlands; ^3^ Department of Genetics, University Medical Centre Groningen University of Groningen Groningen The Netherlands

**Keywords:** eczema, hyperkeratotic hand eczema, immunofluorescence microscopy, keratinocyte differentiation, palmar psoriasis, pathophysiology

## Abstract

**Background:**

Hyperkeratotic hand eczema (HHE) is a typical clinical hand eczema subtype with a largely unknown pathophysiology.

**Objective:**

To investigate histopathology, expression of keratins (K), epidermal barrier proteins, and adhesion molecules in HHE.

**Methods:**

Palmar skin biopsies (lesional and perilesional) were obtained from seven HHE patients and two healthy controls. Moreover, 135 candidate genes associated with palmoplantar keratoderma were screened for mutations.

**Results:**

Immunofluorescence staining showed a significant reduction of K9 and K14 in lesional skin. Upregulation was found for K5, K6, K16, and K17 in lesional skin compared with perilesional and healthy palmar skin. Further, upregulation of involucrin and alternating loricrin staining, both in an extracellular staining pattern, was found. Filaggrin expression was similar in lesional, perilesional, and control skin. No monogenetic mutations were found.

**Conclusion:**

Currently, the phenotype of HHE is included in the hand eczema classification system; however, it can be argued whether this is justified. The evident expression of filaggrin and involucrin in lesional skin does not support a pathogenesis of atopic eczema. The upregulation of K6, K16, and K17 and reduction of K9 and K14 might contribute to the underlying pathogenesis. Unfortunately, comparison with hand eczema studies is not possible yet, because similar protein expression studies are lacking.

## Graphical abstract

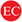

## 1 INTRODUCTION

Hyperkeratotic hand eczema (HHE) is defined by sharply demarcated areas of hyperkeratosis or thick scaling on the palms, possibly extending to the palmar aspects of the fingers.^1^, ^2^ There is little or no redness, and vesicles are absent. Plantar aspects of the feet can be involved as well. There is a male predominance and patients are mainly middle aged.^3^ This phenotype is currently included in the classification system of hand eczema. Two classification systems have been described; however, no consensus has been reached yet. Agner et al consider HHE as an endogenous subtype of hand eczema with no identifiable cause.^4^, ^5^ By contrast, Menné et al use a morphological classification system in which all clinical subtypes, including HHE, could have identifiable causes of hand eczema such as atopic dermatitis (AD), exposure to irritants, or to contact allergens in sensitized persons.^2^ The pathophysiology of this well‐described monomorphic subtype is largely unknown and it could be questioned whether it should be classified as eczema.

Palmar hyperkeratotis is seen in other diseases as well, such as palmar psoriasis and palmar plantar keratoderma (PPK). Palmar psoriasis resembles HHE. It was reported that it can be distinguished from psoriasis by lack of scaling typical for psoriasis, but the clinical distinction is very problematic.^6^ PPK is an umbrella term for any form of persistent thickening of the epidermis at palmar and/or plantar skin and includes both genetic and acquired conditions. Literature about PPK showed that mutations in several keratin genes can result in a weakened cell cytoskeleton resulting in abnormally thickened and keratotic palmoplantar skin.^7^, ^8^ Similarly, mutations in genes encoding desmogleins (DSG‐1‐4), desmocollins (DSC1‐3), desmoplakin (DSP), plakoglobin (PG), plakophilins (PKP1‐3), and corneodesmosin are associated with palmar hyperkeratosis.^9^, ^10^

Here we present a case series of patients with a clinical phenotype of HHE. The aim of this study was to gain more insight into the etiology of this phenotype. Therefore, we have investigated the histopathology, the expression of different palmar keratins, epidermal barrier proteins, and adhesion molecules in lesional and nonlesional skin of patients with the clinical phenotype of HHE. In addition, we screened all patients for variants in genes related to PPK.

## 2 METHODS

### 2.1 Study design and participants

This pilot study was conducted at the Dermatology Department of the University Medical Center Groningen, a tertiary referral center for hand eczema, between August 2018 and December 2018. Adult patients with a clinical phenotype of HHE were eligible.^1^, ^2^ Exclusion criteria were a current diagnosis of AD,^11^ topical corticosteroid use within the last 2 weeks, immunosuppressive/immunomodulatory treatment or ultraviolet radiation therapy within the last 4 weeks, contact allergies with clinical relevance to the hands in which exposure to allergens is not avoided, a history of psoriasis or psoriasis lesions elsewhere on the body, and other (genetic) skin diseases or infections of the hands.

### 2.2 Clinical characteristics

The following data were collected: age; sex; onset of symptoms; history of AD; history of allergic asthma (regarding Global Initiative for Asthma guideline)^12^; history of allergic rhinitis (regarding Allergic Rhinitis and its Impact on Asthma guideline)^13^; specific inhalant immunoglobulin IgE allergens; atopy score <3 points (probability of AD is very small), 3 to 9 points (possible diagnosis of AD), and >10 points (reliable diagnosis of AD)^14^; patch test results; occupation; work‐related exposure to irritants^15^; and foot involvement.

### 2.3 Skin samples

Four biopsies (3 mm) were obtained, two for histopathology and two for immunofluorescence (IF) microscopy from lesional and perilesional (noninflamed) palmar skin of seven patients. In addition, 3‐mm samples from the hypothenar region of the palms of two healthy controls (HCs) were taken for comparison. HC were age and sex matched. The study was ethically approved by the local review board (M18.228998).

### 2.4 Histopathology/immunohistochemistry

Hematoxylin and eosin (H&E)‐stained sections were examined for morphologic features. Epidermal proliferation and differentiation were assessed by immunohistochemical (IHC) staining for Ki‐67‐positive nuclei using anti‐Ki‐67 (30‐9) rabbit monoclonal primary antibody (Ventana Roche, Tucson, Arizona). To analyze the Ki‐67‐positive cells of each section, a line of 1‐mm length following the stratum basale was drawn after choosing a representative region. All positive cells above and under the line were counted by two independent observers (LL and KP) and expressed as “positive cells per millimeter length of basement membrane.”

### 2.5 Immunofluorescence staining

Skin biopsies for IF staining were snap frozen in liquid nitrogen. For IF staining antibodies against K1, K2e, K5, K6, K9, K10, K14, K15, K16, K17, DSG‐1, DSG‐3, plakophilin‐1, plakoglobin, desmoplakin (rod), filaggrin, loricrin, involucrin, and corneodesmosin were used (Table S1). Cryosections of 4‐μm thickness were mounted on Polysine glass slides, air dried for 20 min with a fan, and encircled with a hydrophobic emulsion (PAP pen; Dako, Glostrup, Denmark). The mapping procedure included two consecutive immunostaining steps of 30 min each at ambient temperature, alternating with washing steps with 0.15 mmphosphate‐buffered saline (PBS) for 30 min. The first staining step involved binding of the different monoclonal mouse antigens (Supplementary S1). The second staining step involved fluorescent detection and bound the antibody in step 1. For this purpose we used the highly absorbed Alexa 488‐conjugated goat antimouse IgG (Molecular Probes Europe, Leiden, The Netherlands). Thereafter, a bisbenzimide staining for 5 to 10 minutes to detect the nucleoli and a last washing step in 0.15 mm PBS was performed for 10 to 20 min. The sections were mounted with coverslips under SlowFade antifade. The sections were examined with a Leica DMRA fluorescence microscope and images were acquired by a Leica DFC350 FX digital camera (Leica Microsystems, Bannockburn, Illinois). Further image processing was done by Adobe Photoshop software.

The immunostaining pattern and intensity in the epidermis were examined by three independent observers (MB, HP, and KP). The intensity was scored semiquantitatively using a global assessment on a continuous scale from 0 (negative) to 4 (1+, 2+, 3+, or 4+). The mean scores of the observers were used for the final conclusion.

### 2.6 Gene analysis

DNA was extracted from peripheral blood leukocytes of all patients. Whole‐exome sequencing (Agilent SureSelect Human All Exon V6_S07604514) was performed and a panel of 135 candidate genes associated with palmoplantar keratoderma was screened for relevant variants (Supplementary S2). Gene variants were identified using a standard diagnostic pipeline and variant classification was performed according to criteria described by Fokkema et al and guidelines of the American College of Medical Genetics and Genomics 2015.^16^, ^17^ Because no pathogenic or likely pathogenic mutations were identified in the 135 PPK‐related genes that could be associated with the HHE phenotype, a second analysis was performed. Because all seven patients shared a strikingly similar clinical phenotype, we hypothesized that all patients might carry a variant in the same, as yet unknown, gene. To test this hypothesis an open‐exome cohort analysis was performed. Gene variant analysis was performed using data analysis pipeline to filter out late‐onset genes (eg, *BRCA1*).

## 3 RESULTS

### 3.1 Clinical characteristics

All seven patients were male. Patients had a mean age of onset of 50.7 years (range 38‐61 years) and a mean disease duration of 6.9 years (range 1.5‐20 years; Table 1).

**TABLE 1:** Demographic and clinical characteristics

Patient	Sex	Age^a^	Age of onset^b^	Specific IgE inhalant allergens	AD in childhood	Asthma^12^	Allergic rhinitis^13^	Atopic skin diathesis^14^ ^b^	Positive patch tests	Occupation	Work‐related exposure to irritants	Foot involvement
HHE‐01	M	63	61	−	−	−	−	0	−	Bricklayer	+	–
HHE‐02	M	65	56	−	−	−	−	0	−	Technical service operating rooms	−	+
HHE‐03	M	52	47	+	+	+	+	4	+	Not working	−	–
HHE‐04	M	46	44	+	−	+	+	5	+	Construction worker	+	−
HHE‐05	M	45	38	−	−	–	−	0	+	Accountant	−	–
HHE‐06	M	75	55	+	−	−	+	2	−	Retired (golf player)	−	–
HHE‐07	M	59	54	+	−	−	+	3	+	Process operator (chemical factory)	+	+
HC‐01	M	63	N/A	X	−	−	−	0	X	Photographer	−	–
HC‐02	M	61	N/A	X	−	–	−	0	X	Construction worker	+	−

*Note*: Clinical features of seven patients with a clinical phenotype of hyperkeratotic hand eczema (HHE) and two healthy controls (HCs).

Abbreviations: AD, atopic dermatitis; Ig, immunoglobulin; M, male; N/A, not applicable; X, not performed; +, yes; –, no.

a Age in years.

b Atopy score: < 3 points = probability of AD is very small, ≥ 3‐9 points = possible diagnosis of AD.

**FIGURE 1:**
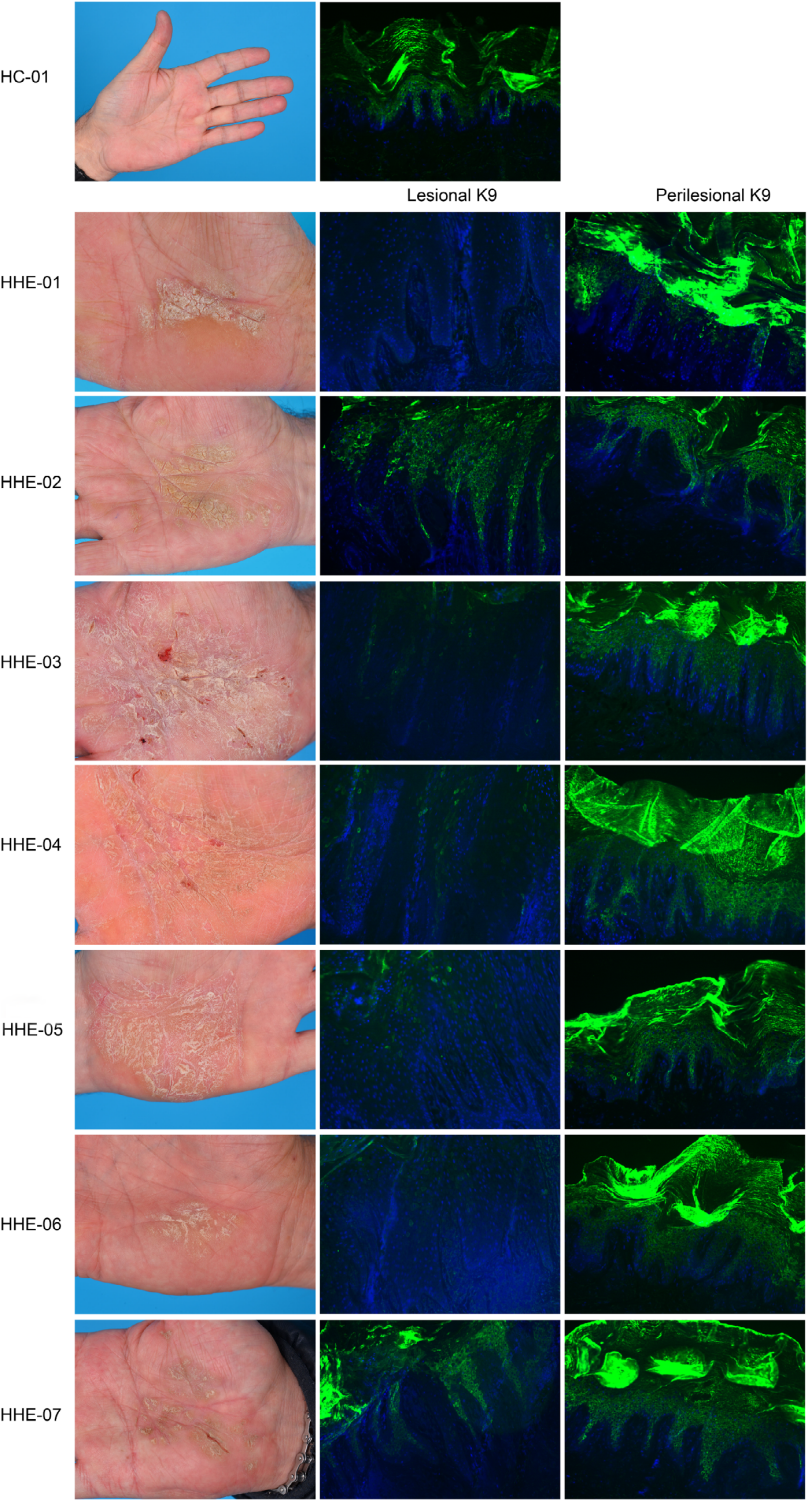
In the left column, clinical pictures showing the palms of the seven patients with a clinical phenotype of hyperkeratotic hand eczema. The two columns at the right show the K9 immunofluorescence staining patterns of their skin biopsies: reduced or absent staining of keratin 9 in lesional skin (5/7, middle column) and normal K9 staining in perilesional (7/7, right column) and healthy control (HC) skin

All patients showed a consistent clinical picture with sharply demarcated hyperkeratosis on the palms with painful fissures, and no or mild redness (Figure 1). Two patients had concomitant plantar involvement consisting of hyperkeratotic plaques without erythema. None of the patients had current AD and one patient had a history of AD in childhood. In four of seven patients elevated levels of specific IgE inhalation allergens were found. Two patients had asthma and four patients had (mild) symptoms of allergic rhinitis. The atopy score showed a possible atopic skin diathesis in three patients. Three of seven patients had an irritant exposure, mostly due to work‐related mechanical stress and/or chemical irritants. None of the patients had a family history of palmoplantar keratoderma or psoriasis. Six patients had a previous history of systemic treatment, frequently alitretinoin, acitretin, and methotrexate.

### 3.2 Histopathology

All lesional skin biopsies presented the following histopathologic features in H&E‐stained sections: confluent parakeratosis, plasma in the parakeratotic foci, presence of the stratum granulosum, epidermal hyperplasia, spongiosis, and exocytosis of lymphocytes. Typical characteristics of palmar psoriasis (eg, neutrophilic granulocytes in parakeratotic foci and absence of a granular layer) were absent in all biopsies. In five of seven perilesional skin biopsies mild signs of inflammation were found: a sparse superficial perivascular infiltrate of lymphocytes without spongiotic and hyperkeratotic changes.

### 3.3 Epidermal proliferation

IHC analysis showed a significantly higher number of Ki‐67‐positive nuclei per millimeter basement membrane (mean ± standard deviation) in lesional skin (279 ± 75) compared with perilesional skin (75 ± 28; one perilesional Ki‐67 staining was missing) and HC skin (51 ± 6). The number of Ki‐67‐positive nuclei in perilesional skin was 27% of that in lesional skin (*P* < .05).

### 3.4 Immunofluorescence staining

**FIGURE 2:**
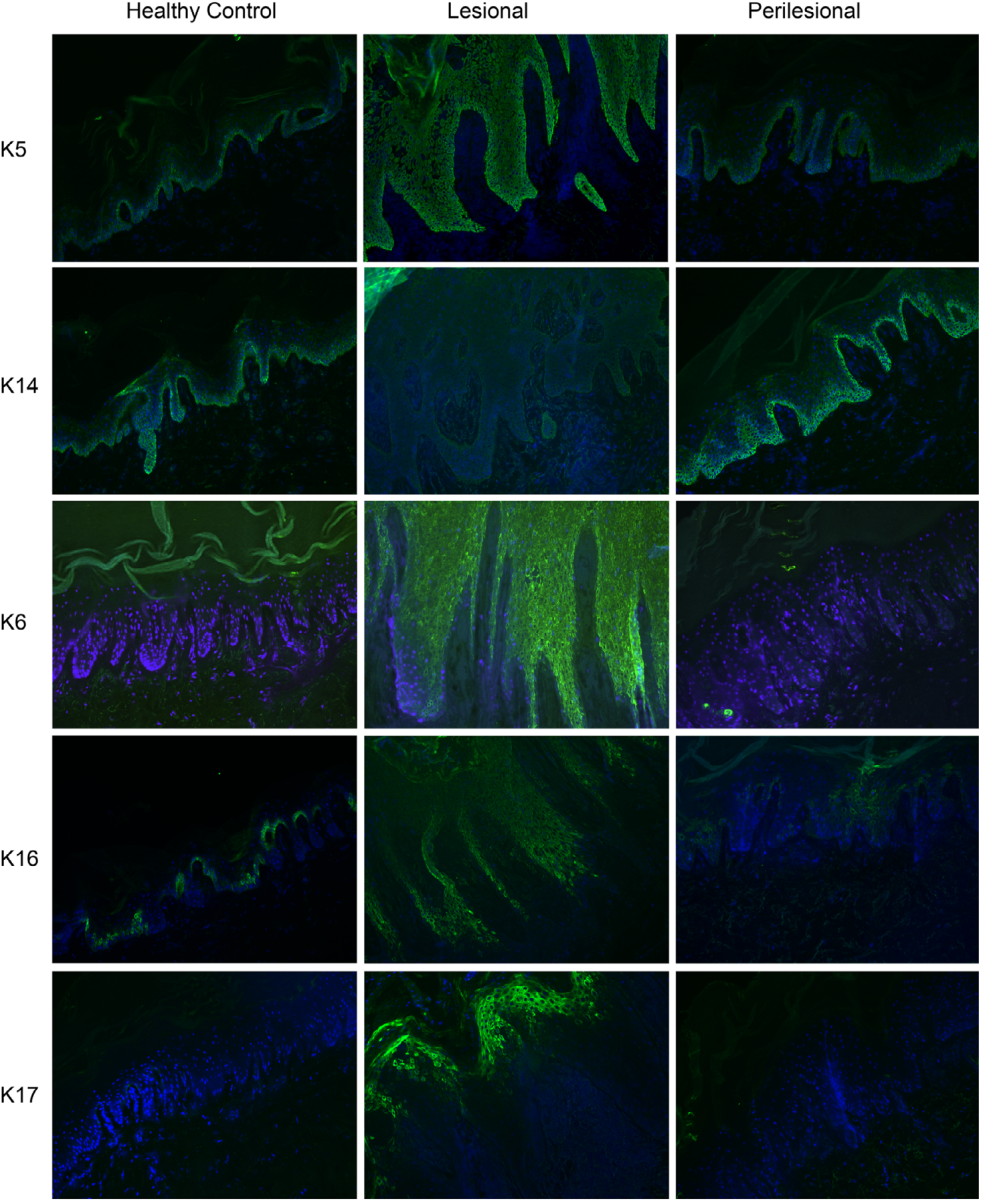
Immunofluorescence staining pattern of keratin (K) 5, 14, 6, 16, 17 in healthy control (HC), lesional, and perilesional skin of hyperkeratotic hand eczema patients. K5, K6, K16, and K17 were strongly upregulated in lesional skin compared with perilesional and HC skin. K14 was downregulated in lesional skin compared with perilesional and HC skin

K5 showed an intense panepidermal staining in six of seven lesional skin samples. Perilesional and HC skin presented normal K5 basal cell staining. K14 staining was strongly reduced in all lesional skin samples and two of seven perilesional skin samples. A normal basal K14 staining was found in five of seven perilesional and both HC skin samples (Figure 2). K15 was negative in palmar lesional, perilesional, and HC skin.

The diffuse staining of K1 and K10 in the suprabasal layers was comparable between lesional, perilesional, and HC skin. Furthermore, K2e staining in the upper spinous layer was similar in all groups. K9 was significantly decreased or even absent in five of seven lesional skin biopsies compared with normal suprabasal staining in all perilesional and HC skin samples (Figure 1). Two patients had normal K9 staining in lesional skin.

An evident panepidermal upregulation of K6 was found in all lesional skin samples. Some patches of positive basal and/or suprabasal staining were found in six of seven perilesional skin samples. Both HC skin samples were negative for K6. K16 staining presented a strong panepidermal staining in five of seven lesional skin samples compared with a basal layer‐limited staining in HC. In two of seven lesional and two of seven perilesional skin samples a dubious K16 staining was found. In five of seven perilesional skin samples a mainly basal and some suprabasal staining was seen. K17 was strongly positive in parts of the suprabasal layers in all lesional skin biopsies. Perilesional and HC skin samples were negative for K17 (Figure 2).

**FIGURE 3:**
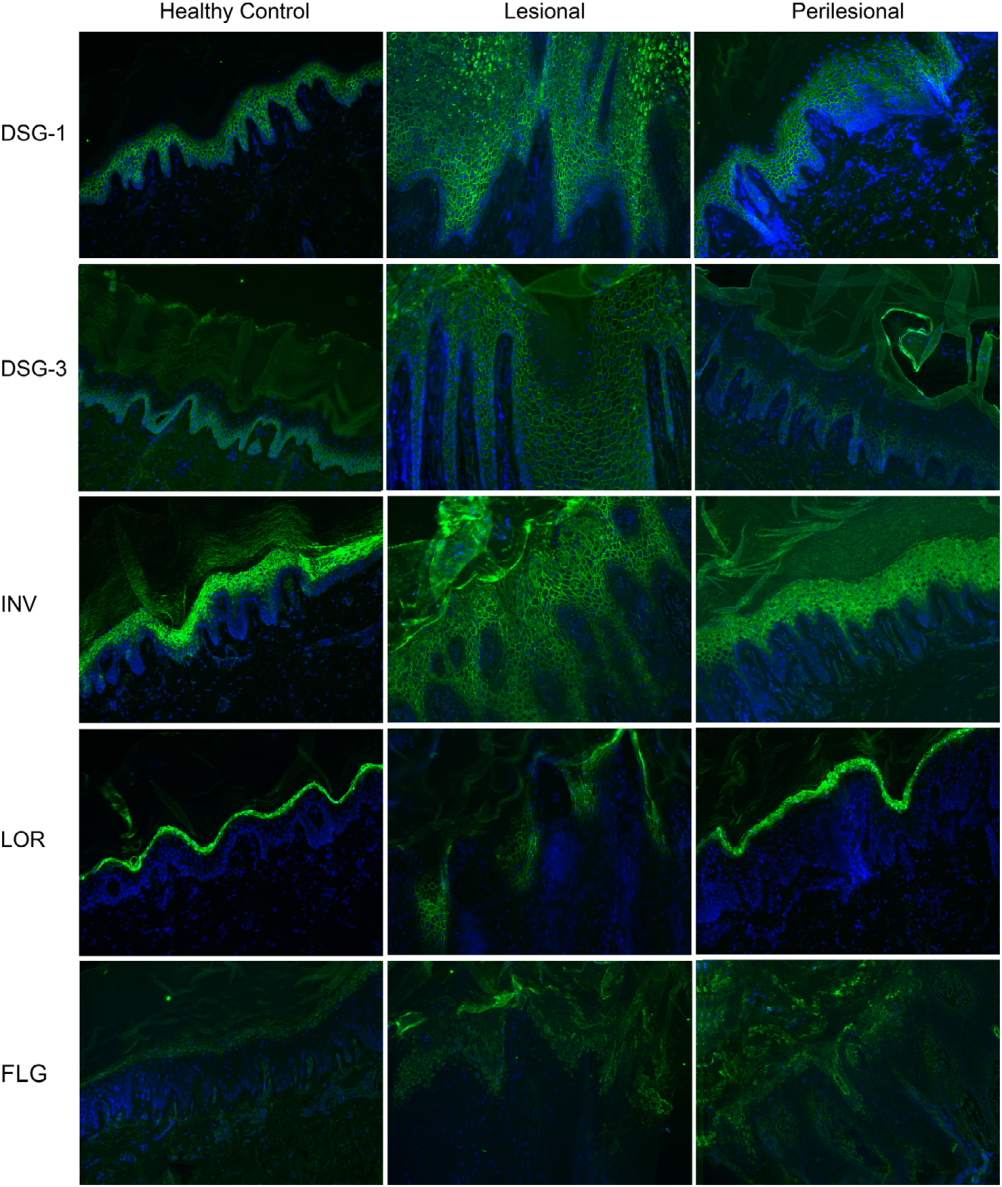
Immunofluorescence pattern of desmoglein (DSG) 1 and 3, involucrin (INV), loricrin (LOR), and filaggrin (FLG) in healthy control (HC), lesional, and perilesional skin of hyperkeratotic hand eczema patients. DSG‐1 staining in lesional skins showed certain keratinocytes with an intracellular staining compared with the normal extracellular staining in perilesional and HC skin. In DSG‐3 more panepidermal staining was found in lesional skin. Involucrin showed an extracellular and earlier staining in lesional skin compared with perilesional and HC skin. Loricrin staining showed alternating extracellular staining compared with intracellular stratum granulosum staining in perilesional and HC skin. Filaggrin was similarly expressed in the stratum granulosum

DSG‐1 staining showed a normal epidermal cell surface (ECS) staining in all groups. However, in all lesional and some perilesional skin samples, three of seven certain keratinocytes were found with a granular intracellular staining in the stratum granulosum. For DSG‐3 a more panepidermal ECS pattern was seen in lesional skin compared with perilesional and HC skin (Figure 3). Staining with antibodies against plakophilin‐1, plakoglobin, and desmoplakin showed normal ECS staining in lesional compared with perilesional and HC skin (data not shown).

Involucrin staining in lesional skin showed an earlier than normal expression in a mainly ECS pattern in the complete spinous layer. Perilesional and HC skin samples showed a granular intracellular reactivity of involucrin in the upper spinous layers which gradually increased in ascending layers. Lesional loricrin staining showed, in five of seven skin samples, alternation of positive patches of loricrin staining in an ECS pattern in the stratum granulosum. In perilesional and HC skin normal cytoplasmic staining was found in the upper spinous layers which gradually increased in ascending layers (Figure 3). Filaggrin staining was found in the horny and granular layers of all skin samples; also lesional skin showed normal levels of filaggrin expression. In lesional skin, the expression of filaggrin was seen in more layers, due to acanthosis. Corneodesmosin showed normal ECS staining in the stratum corneum of all biopsies.

### 3.5 Gene analysis

Analysis of the 135 PPK‐related genes in the seven patients did not identify any pathogenic or likely pathogenic variants that could be associated with the HHE phenotype. In total, three pathogenic mutations and four variants of unknown significance (VOUS) were identified. These heterozygous variants were identified in genes for autosomal recessive skin disorders, and could be easily excluded as causative for the HHE phenotype on the basis of inheritance and absence of related phenotypic features. Particularly, no mutations in epidermal barrier genes such as filaggrin (*FLG*)*,* Filaggrin‐2 (*FLG2*), and serine protease inhibitor Kazal type 5 (*SPINK5*) were detected. In addition, the cohort‐analysis did not reveal a single mutated gene (Supplementary S3).

## 4 DISCUSSION

In this pilot study we have assessed the underlying etiology of the clinical phenotype of HHE. Our main findings were identical histopathological features in all lesional skin biopsies, an epidermal hyperproliferation, and abnormal differentiation with strong K5, K6, K16, K17 and reduced K14 and K9 staining, and altered DSG‐1, DSG‐3, involucrin, and loricrin staining patterns. The main question in this study was whether our findings support a diagnosis of HHE. Therefore, we will discuss our most evident findings in comparison with the literature on hand eczema and hyperkeratotic palmar diseases such as palmar psoriasis and PPK.

In general, the pathogenesis of hand eczema remains largely unclear and is seen as a complex interaction between endogenous and exogenous factors. An exogenous factor for developing hand eczema is repeated contact with irritating agents and factors (friction).^1^ Irritants induce a disruption of the skin barrier, and lead to an inflammatory reaction, mainly mediated by the innate immunity.^18^ K6/16/17 are early barrier alarmins and upregulation of these keratins is seen in both wounding and exposure to irritants, which results in keratinocyte hyperproliferation and hyperkeratosis.^19^ Irritants induce also an early expression of involucrin.^20^, ^21^ Similar results were found in our study. However, this could not sufficiently cover the whole etiology because not all patients with HHE had exposure to irritants. Probably, HHE patients have a pre‐existent skin barrier problem where exposure to irritating agents and factors eventually leads to secondary dysregulation of the immune system.^22^ The skin barrier problem is an underlying etiology in AD.^22^, ^23^ A two‐ to four‐fold increased risk to develop hand eczema was found in patients with a previous or current history of AD.^24^ However, in the current study only one patient had a history of AD in childhood. Three patients had a possible atopic skin diathesis based on the atopy score.^14^ Four patients had mucosal atopy, although previous studies showed no association between mucosal atopy and an increased risk of developing hand eczema.^25^, ^26^, ^27^

The histopathology of all lesional skin biopsies in this study presented confluent parakeratosis, plasma in the parakeratotic foci, presence of the stratum granulosum, epidermal hyperplasia, and spongiosis. These morphological features were described in HHE before.^28^ However, Park et al described the histological changes in biopsies of 25 HHE patients and 16 patients with palmar psoriasis and found no significant differences.^29^ It is therefore questionable if differentiation based on histopathology is possible. Summarizing, both histopathology and contributing etiological factors, such as exposure to irritants and a history of atopic eczema, could not completely explain the pathogenesis of this clinical phenotype.

Circumscribed hyperkeratosis of the palms is also seen in palmar psoriasis. The clinical distinction between HHE and palmoplantar psoriasis is difficult when plaques are only located on the palms.^29^, ^30^, ^31^ Only few studies have investigated both diseases.^29^, ^30^, ^32^, ^33^ Lillis et al investigated interleukin‐23 (IL‐23), which stimulates T helper 17 (Th17) cell survival and proliferation, and found a significant upregulation of IL‐23 which did not significantly differ between HHE and (palmar) psoriasis.^30^ Possibly, the inflammation in the clinical phenotype of HHE is, as psoriasis, more Th17 driven. This is also supported by the strong lesional K17 staining in our study which plays an important role in the pathogenesis of psoriasis.^19^, ^34^, ^35^ K17 is a stimulator of psoriatic T cells (Th‐17) and producer of specific cytokines (IL‐17) which in turn stimulate K17, again leading to an “autoimmune feedback loop.”^36^, ^37^ This “feedback loop” might explain the persistent palmar hyperkeratosis at the same location of this clinical phenotype. The persistent plaques were also described by Hersle and Mobacken, who noticed after a follow‐up of 10 years an almost identical clinical monomorphic picture.^31^ By contrast, other clinical subtypes of hand eczema, as well as AD, show more polymorphic skin lesions which tend to vary in appearance and location over time.^1^, ^11^, ^38^ Future studies should evaluate whether the phenotype of HHE is a variant of (localized) psoriasis.

Furthermore, a common denominator in the pathophysiology of chronic inflammatory skin diseases such as AD, hand eczema, and psoriasis seems to be skin barrier impairment.^39^, ^40^, ^41^ We studied involucrin, loricrin, and filaggrin because these proteins have a major role in forming the cornified envelope. Involucrin provides a scaffold to which other proteins, such as loricrin, subsequently cross‐link to form the cornified envelope.^42^ Filaggrin contributes herein by, among others, aggregating keratin filaments and organizing lipid bilayers.^43^ Mutations in *FLG* are known to be a risk factor for the development of AD. An independent association of *FLG* mutations and hand eczema has not been found.^44^, ^45^, ^46^, ^47^ Furthermore, independently of gene mutations, studies in AD showed lower expression levels of filaggrin, involucrin, and loricrin due to proinflammatory cytokines such as IL‐4.^48^, ^49^ Both *FLG* mutations and decreased filaggrin expression were not found in the current study. In two other studies filaggrin expression in hand eczema biopsies was analyzed. Molin et al analyzed the whole proteome by using liquid chromatography with tandem mass spectrometry analysis of palmar skin biopsies from six morphologically unclassified hand eczema patients.^50^ Subsequently, IHC was performed in seven other hand eczema patients to confirm the results. Another study in patients with hyperkeratotic fissured hand eczema by Kumari et al analyzed filaggrin and loricrin by fluorescence‐based real‐time quantitative polymerase chain reaction (qPCR), and subsequently IHC in 15 skin biopsies.^40^ Both studies showed that filaggrin expression was downregulated. *FLG* mutations were not analyzed in both studies. Morphological subtypes of hand eczema were not clearly described in these studies. A possible explanation for the differences between our study and the previous studies might be the different patient populations with different morphological subtypes of hand eczema. In the current study we included patients with a typical phenotype of HHE with stable plaques and less redness and no other signs of inflammation. Other morphological subtypes of hand eczema with obvious inflammation may lead to a secondary downregulation of filaggrin expression. The question is whether the differences between this study and the previous two studies, with respect to expression of epidermal barrier proteins, can be explained by different morphological types of hand eczema. It could also be considered whether HHE is a subtype of hand eczema or has another etiology. Besides AD and hand eczema, Kim et al studied filaggrin, involucrin, and loricrin in skin biopsies of 10 adult patients with psoriasis. qPCR and IHC showed significantly decreased filaggrin and loricrin expression; however, involucrin was not decreased in lesional skin. An interesting finding in this study was that tumor necrosis factor‐α (TNF‐α)‐stimulated keratinocytes showed only reduced filaggrin and loricrin expression. In contrast to TNF‐α, keratinocytes stimulated by Th2 cytokines showed reduced expression of filaggrin, loricrin, and also involucrin.^41^ In our study involucrin was strongly presented in an ECS staining pattern through the complete spinous layer. Loricrin was downregulated and alternately presented in an ECS pattern. Perilesional and HC skin showed unaltered cytoplasmic staining for both in the upper stratum spinous and granular layers. The early expression of involucrin was described in other hyperproliferative diseases, such as psoriasis and PPK, but an ECS staining pattern for both proteins has not been described before.^21^, ^51^, ^52^ We hypothesize that the ECS staining pattern of involucrin and loricrin might be explained by an increased keratinocyte proliferation, whereby the cornified envelope is prematurely formed and involucrin and loricrin are earlier attached underneath the plasma membrane instead of the cytoplasm. Another remarkable finding in our study was the upregulation of involucrin, which has not been described in hand eczema before, although sufficient studies are lacking. The opposite, a downregulation of involucrin, is described in AD. Therefore, possibly other cytokine profiles result in palmar hyperkeratosis in HHE.^40^, ^41^, ^48^, ^50^, ^53^

Hereditary PPK is another group of skin diseases with palmar hyperkeratosis, which provides more insight about the association of different keratins, desmosomal proteins, and epidermal barrier proteins with palmoplantar hyperkeratosis.^8^, ^9^, ^10^, ^54^, ^55^, ^56^, ^57^, ^58^ In this study no monogenetic mutations related to palmar hyperkeratosis were found. However, a contributing role for one of the VOUS cannot be completely excluded.

Despite not being able to identify gene mutations, this study showed abnormal differentiation in important keratins. K5 was upregulated and K14 and K9 staining were downregulated in lesional skin compared with HCs. Only a few small studies describe keratin expression in other palmar skin diseases and found contradicting results.^59^, ^60^ Normally, K9 is only expressed in healthy ridged skin. In the current study, lesional palmar skin had lost K9 expression, which might indicate that palmar keratinocytes can change their specific pathways to a trunk‐type keratinization. Beside keratins, desmosomes also have an important function in epidermal differentiation. Normally, DSG‐3 has a strong basal distribution associated with the proliferating cells, whereas DSG‐1 expression increases from the basal to the granular layer. However, DSG‐1 and DSG‐3 staining in this study were both panepidermally expressed in lesional skin and showed remarkable granular staining for DSG‐1. Desmogleins are in continuous turnover in the desmosome and are discarded by cellular uptake to be destroyed or recycled.^61^ Perhaps the abnormal differentiation and disturbed forming of the cornified envelope results in a higher DSG‐1 turnover and uptake, whereby DSG‐1 is not only expressed in the membrane but also in the cytoplasm. Further research should elucidate whether the abnormal desmosomal and keratin staining represent a primary defect in the pathophysiology of HHE, or are of secondary nature.

One of the limitations of this study is the relatively small study sample, though clinical presentations were very uniform. Another limitation is that IF staining is a strong diagnostic method in case of evident differences, while smaller differences can be missed. Furthermore, it is difficult to compare our IF data with previous literature on other palmar skin diseases, as most studies focused on nonridged skin which differs from ridged skin we assessed.^62^, ^63^ Finally, we did not include other hyperkeratotic palmar diseases as palmar psoriasis and PPK in this study. Future studies should investigate disease‐specific biomarkers, such as NOS2 (psoriasis) and CCL27 (eczema) in different palmar skin diseases.^64^

In conclusion, the phenotype of HHE is currently included in the classification system of hand eczema; however, it can be argued whether this is justified. The evident expression of filaggrin and involucrin in lesional skin does not support a pathogenesis of atopic eczema. The upregulation of K6, K16, and K17 and the contrasting reduction or even absence of K9 and K14 might contribute to the underlying pathogenesis. Unfortunately, comparison with hand eczema studies is not possible because similar protein expression studies are lacking. Further studies are necessary to compare our results with other subtypes of hand eczema and other palmoplantar skin diseases to see which pathways are involved.

## CONFLICT OF INTEREST

There was no funding and the authors have no conflicts either actual or perceived.

## Supporting information


**Supplementary S1** Supporting information.

**Supplementary S2** Supporting information.

**Supplementary S3** Supporting information.

## References

[1] Coenraads PJ . Hand eczema. N Engl J Med. 2012;367(19):1829‐1837. 23134383 10.1056/NEJMcp1104084

[2] Menné T , Johansen JD , Sommerlund M , Veien NK . Hand eczema guidelines based on the Danish guidelines for the diagnosis and treatment of hand eczema. Contact Dermatitis. 2011;65(1):3‐12. 21658053 10.1111/j.1600-0536.2011.01915.x

[3] van der Heiden J , Agner T , Rustemeyer T , Clemmensen KKB . Hyperkeratotic hand eczema compared to other subgroups of hand eczema – a retrospective study with a follow‐up questionnaire. Contact Dermatitis. 2018;78(3):216‐222. 29314088 10.1111/cod.12945

[4] Diepgen TL , Andersen KE , Brandão FM , et al. Hand eczema classification: a cross‐sectional, multicentre study of the aetiology and morphology of hand eczema. Br J Dermatol. 2009;160(2):353‐358. 19016702 10.1111/j.1365-2133.2008.08907.x

[5] Agner T , Aalto‐Korte K , Andersen KE , et al. Classification of hand eczema. J Eur Acad Dermatol Venereol. 2015;29(12):2417‐2422. 26371368 10.1111/jdv.13308

[6] Johansen JD , Hald M , Andersen BL , et al. Classification of hand eczema: clinical and aetiological types. Based on the guideline of the Danish Contact Dermatitis Group. Contact Dermatitis. 2011;65(1):13‐21. 21658054 10.1111/j.1600-0536.2011.01911.x

[7] Moll R , Divo M , Langbein L . The human keratins: biology and pathology. Histochem Cell Biol. 2008;129(6):705‐733. 18461349 10.1007/s00418-008-0435-6 PMC2386534

[8] Guerra L , Castori M , Didona B , Castiglia D , Zambruno G . Hereditary palmoplantar keratodermas. Part I. non‐syndromic palmoplantar keratodermas: classification, clinical and genetic features. J Eur Acad Dermatol Venereol. 2018;32(5):704‐719. 29489036 10.1111/jdv.14902

[9] Petrof G , Mellerio JE , McGrath JA . Desmosomal genodermatoses. Br J Dermatol. 2012;166(1):36‐45. 21929534 10.1111/j.1365-2133.2011.10640.x

[10] Ishida‐Yamamoto A , Igawa S . Genetic skin diseases related to desmosomes and corneodesmosomes. J Dermatol Sci. 2014;74(2):99‐105. 24636350 10.1016/j.jdermsci.2014.02.005

[11] Williams HC , Burney PG , Pembroke AC , Hay RJ . Validation of the U.K. diagnostic criteria for atopic dermatitis in a population setting. U.K. diagnostic criteria for atopic dermatitis working party. Br J Dermatol. 1996 Jul;135(1):12‐17. 8776351

[12] Global Initiative for Asthma . Global stragey for asthma management and prevention. www.ginasthma.org. Accessed November 2019.

[13] Bousquet J , Khaltaev N , Cruz AA , et al. An introduction of allergic rhinitis and its impact on asthma (ARIA) 2008 update. Allergy. 2008;63(86):8‐160. 18331513 10.1111/j.1398-9995.2007.01620.x

[14] Diepgen TL , Sauerbrei W , Fartasch M . Development and validation of diagnostic scores for atopic dermatitis incorporating criteria of data quality and practical usefulness. J Clin Epidemiol. 1996;49(9):1031‐1038. 8780613 10.1016/0895-4356(96)00119-9

[15] Cazzaniga S , Apfelbacher C , Diepgen T , et al. Patterns of chronic hand eczema: a semantic map analysis of the CARPE registry data. Br J Dermatol. 2018;178(1):229‐237. 28498524 10.1111/bjd.15660

[16] Fokkema IFAC , Velde KJ , Slofstra MK , et al. Dutch genome diagnostic laboratories accelerated and improved variant interpretation and increased accuracy by sharing data. Hum Mutat. 2019;40(12):2230‐2238. 10.1002/humu.23896 31433103 PMC6900155

[17] Richards S , Aziz N , Bale S , et al. Standards and guidelines for the interpretation of sequence variants: a joint consensus recommendation of the American College of Medical Genetics and Genomics and the Association for Molecular Pathology. Genet Med. 2015;17(5):405‐424. 25741868 10.1038/gim.2015.30 PMC4544753

[18] Jakasa I , Thyssen JP , Kezic S . The role of skin barrier in occupational contact dermatitis. Exp Dermatol. 2018;27(8):909‐914. 29894020 10.1111/exd.13704

[19] Zhang X , Yin M , Zhang L . Keratin 6, 16 and 17—critical barrier alarmin molecules in skin wounds and psoriasis. Cell. 2019;8(8):807. 10.3390/cells8080807 PMC6721482 31374826

[20] Mai Le TK , Schalkwijk J , van de Kerkhof PCM , van Haelst U , van der Valk PGM . A histological and immunohistochemical study on chronic irritant contact dermatitis. Am J Contact Dermat. 1998;9(1):23‐28. 9471983

[21] Ekanayake‐Mudiyanselage S , Aschauer H , Schmook FP , Jensen JM , Meingassner JG , Proksch E . Expression of epidermal keratins and the cornified envelope protein involucrin is influenced by permeability barrier disruption. J Invest Dermatol. 1998;111(3):517‐523. 10.1046/j.1523-1747.1998.00318.x 9740250

[22] Kezic S . Atopic dermatitis: risk estimates for hand eczema. Br J Dermatol. 2018;178(4):827. 10.1111/bjd.16343 29668088

[23] Han H , Roan F , Ziegler SF . The atopic march: current insights into skin barrier dysfunction and epithelial cell‐derived cytokines. Immunol Rev. 2017;278(1):116‐130. 28658558 10.1111/imr.12546 PMC5492959

[24] Ruff SMD , Engebretsen KA , Zachariae C , et al. The association between atopic dermatitis and hand eczema: a systematic review and meta‐analysis. Br J Dermatol. 2017;178(4):879‐888. 10.1111/bjd.16147 29172235

[25] Grönhagen C , Lidén C , Wahlgren CF , et al. Hand eczema and atopic dermatitis in adolescents: a prospective cohort study from the BAMSE project. Br J Dermatol. 2015;173(5):1175‐1182. 26152456 10.1111/bjd.14019

[26] Mortz CG , Lauritsen JM , Bindslev‐Jensen C , Andersen KE . Prevalence of atopic dermatitis, asthma, allergic rhinitis, and hand and contact dermatitis in adolescents. The Odense adolescence cohort study on atopic diseases and dermatitis. Br J Dermatol. 2001;144(3):523‐532. 11260009 10.1046/j.1365-2133.2001.04078.x

[27] Meding B , Jarvholm B . Incidence of hand eczema‐apopulation‐based retrospective study. J Invest Dermatol. 2004;122(4):873‐877. 15102075 10.1111/j.0022-202X.2004.22406.x

[28] Weedon D. Weedon's Skin Pathology 3rd edition. Churchill Livingstone; 2010 pp. 114‐115.

[29] Park JY , Cho EB , Park EJ , Park HR , Kim KH , Kim KJ . The histopathological differentiation between palmar psoriasis and hand eczema: a retrospective review of 96 cases. J Am Acad Dermatol. 2017;77(1):130‐135. 28190621 10.1016/j.jaad.2017.01.005

[30] Lillis JV , Guo C‐S , Lee JJ , Blauvelt A . Increased IL‐23 expression in palmoplantar psoriasis and hyperkeratotic hand dermatitis. Arch Dermatol. 2010;146(8):918‐919. 20713832 10.1001/archdermatol.2010.168

[31] Hersle K , Mobacken H . Hyperkeratotic dermatitis of the palms. Br J Dermatol. 1982;107(2):195‐201. 6213253 10.1111/j.1365-2133.1982.tb00338.x

[32] Errichetti E , Stinco G . Dermoscopy in differential diagnosis of palmar psoriasis and chronic hand eczema. J Dermatol. 2016;43(4):423‐425. 26460228 10.1111/1346-8138.13142

[33] Kolesnik M , Franke I , Lux A , Quist SR , Gollnick HP . Eczema in psoriatico: an important differential diagnosis between chronic allergic contact dermatitis and psoriasis in palmoplantar localization. Acta Derm Venereol. 2018;98(1):50‐58. 28853491 10.2340/00015555-2779

[34] Leigh IM , Navsaria H , Purkis PE , McKay IA , Bowden PE , Riddle PN . Keratins (K16 and K17) as markers of keratinocyte hyperproliferation in psoriasis in vivo and in vitro. Br J Dermatol. 1995;133(4):501‐511. 7577575 10.1111/j.1365-2133.1995.tb02696.x

[35] Bovenschen HJ , Seyger MMB , Van De Kerkhof PCM . Plaque psoriasis vs. atopic dermatitis and lichen planus: a comparison for lesional T‐cell subsets, epidermal proliferation and differentiation. Br J Dermatol. 2005;153(1):72‐78. 16029329 10.1111/j.1365-2133.2005.06538.x

[36] Yang L , Zhang S , Wang G . Keratin 17 in disease pathogenesis: from cancer to dermatoses. J Pathol. 2019;247(2):158‐165. 30306595 10.1002/path.5178

[37] Shi X , Jin L , Dang E , et al. IL‐17A upregulates keratin 17 expression in keratinocytes through STAT1‐andSTAT3‐dependent mechanisms. J Invest Dermatol. 2011;131(12):2401‐2408. 21796151 10.1038/jid.2011.222

[38] Guttman‐Yassky E , Krueger JG . Atopic dermatitis and psoriasis: two different immune diseases or one spectrum? Curr Opin Immunol. 2017;48:68‐73. 28869867 10.1016/j.coi.2017.08.008

[39] Molin S . Pathogenese des Handekzems [Pathogenesis of hand eczema]. Hautarzt. 2019;70(10):755‐759. 31501971 10.1007/s00105-019-04474-5

[40] Kumari V , Timm K , Kühl AA , Heine G , Worm M . Impact of systemic alitretinoin treatment on skin barrier gene and protein expression in patients with chronic hand eczema. Br J Dermatol. 2016;175(6):1243‐1250. 27480504 10.1111/bjd.14921

[41] Kim BE , Howell MD , Guttman E , et al. TNF‐α downregulates filaggrin and loricrin through c‐JunN‐terminal kinase: role for TNF‐α antagonists to improve skin barrier. J Invest Dermatol. 2011;131(6):1272‐1279. 21346775 10.1038/jid.2011.24 PMC8609659

[42] Candi E , Schmidt R , Melino G . The cornified envelope: a model of cell death in the skin. Nat Rev Mol Cell Biol. 2005;6(4):328‐340. 15803139 10.1038/nrm1619

[43] Brown SJ , McLean WHI . One remarkable molecule: filaggrin. J Invest Dermatol. 2012;132(3 PART 2):751‐762. 22158554 10.1038/jid.2011.393 PMC3378480

[44] Lerbaek A , Bisgaard H , Agner T , Ohm Kyvik K , Palmer CNA , Menné T . Filaggrin null alleles are not associated with hand eczema or contact allergy. Br J Dermatol. 2007;157(6):1199‐1204. 17970802 10.1111/j.1365-2133.2007.08252.x

[45] Visser MJ , Verberk MM , Campbell LE , et al. Filaggrin loss‐of‐function mutations and atopic dermatitis as risk factors for hand eczema in apprentice nurses: Part II of a prospective cohort study. Contact Dermatitis. 2014;70(3):139‐150. 24102300 10.1111/cod.12139 PMC4357292

[46] Visser MJ , Landeck L , Campbell LE , et al. Impact of atopic dermatitis and loss‐of‐function mutations in the filaggrin gene on the development of occupational irritant contact dermatitis. Br J Dermatol. 2013;168(2):326‐332. 23039796 10.1111/bjd.12083 PMC3974545

[47] Palmer CN , Irvine AD , Terron‐Kwiatkowski A , et al. Common loss‐of‐function variants of the epidermal barrier protein filaggrin are a major predisposing factor for atopic dermatitis. Nat Genet. 2006;38(4):441‐446. 16550169 10.1038/ng1767

[48] Kim BE , Leung DJM , Boguniewicz M , Howell MD . Loricrin and involucrin expression is down‐regulated by Th2 cytokines through STAT‐6. Clin Immunol. 2008;126(3):332‐337. 18166499 10.1016/j.clim.2007.11.006 PMC2275206

[49] Bao L , Mohan GC , Alexander JB , et al. A molecular mechanism for IL‐4 suppression of loricrin transcription in epidermal keratinocytes: implication for atopic dermatitis pathogenesis. Innate Immun. 2017;23(8):641‐647. 28952836 10.1177/1753425917732823

[50] Molin S , Merl J , Dietrich KA , et al. The hand eczema proteome: imbalance of epidermal barrier proteins. Br J Dermatol. 2015;172(4):994‐1001. 25244099 10.1111/bjd.13418

[51] Wan H , Dopping‐Hepenstal PJC , Gratian MJ , et al. Striate palmoplantar keratoderma arising from desmoplakin and desmoglein 1 mutations is associated with contrasting perturbations of desmosomes and the keratin filament network. Br J Dermatol. 2004;150(5):878‐891. 15149499 10.1111/j.1365-2133.2004.05996.x

[52] Chen JQ , Man XY , Li W , et al. Regulation of involucrin in psoriatic epidermal keratinocytes: the roles of ERK1/2 and GSK‐3β. Cell Biochem Biophys. 2013;66(3):523‐528. 23283814 10.1007/s12013-012-9499-y

[53] Danso MO , Van Drongelen V , Mulder A , et al. TNF‐α and Th2 cytokines induce atopic dermatitis‐like features on epidermal differentiation proteins and stratum corneum lipids in human skin equivalents. J Invest Dermatol. 2014;134(7):1941‐1950. 24518171 10.1038/jid.2014.83

[54] Shemanko CS , Mellerio JE , Tidman MJ , Lane EB , Eady RAJ . Severe palmo‐plantar hyperkeratosis in Dowling‐Meara epidermolysis bullosa simplex caused by a mutation in the keratin 14 gene (KRT14). J Invest Dermatol. 1998;111(5):893‐895. 9804355 10.1046/j.1523-1747.1998.00388.x

[55] Livingston RJ , Sybert VP , Smith LT , Dale BA , Presland RB , Stephens K . Expression of a truncated keratin 5 may contribute to severe palmar‐plantar hyperkeratosis in epidermolysis bullosa simplex patients. J Invest Dermatol. 2001;116(6):970‐974. 11407989 10.1046/j.1523-1747.2001.01324.x

[56] Ishida‐Yamamoto A . Loricrin keratoderma: a novel disease entity characterized by nuclear accumulation of mutant loricrin. J Dermatol Sci. 2003;31(1):3‐8. 12615358 10.1016/s0923-1811(02)00143-3

[57] Liu X , Qiu C , He R , Zhang Y , Zhao Y. Keratin 9 L164P mutation in a Chinese pedigree with epidermolytic palmoplantar keratoderma, cytokeratin analysis, and literature review. Mol Genet Genomic Med. 2019;7(11):e977. 31525823 10.1002/mgg3.977 PMC6825865

[58] Fu DJ , Thomson C , Lunny DP , et al. Keratin 9 is required for the structural integrity and terminal differentiation of the palmoplantar epidermis. J Invest Dermatol. 2014;134(3):754‐763. 23962810 10.1038/jid.2013.356 PMC3923277

[59] Peryassu MA , Cotta‐Pereira G , Ramos‐E‐Silva M , Filgueira AL . Expression of keratins 14, 10 and 16 in marginal keratoderma of the palms. Acta Dermatovener Cr. 2005;13(4):206‐211. 16356392

[60] Tanioka M , Miyagawa‐Hayashino A , Manabe T , Toichi E , Miyachi Y , Takahashi K . Circumscribed palmo‐plantar hypokeratosis: a disease with two subtypes. J Invest Dermatol. 2009;129(4):1045‐1047. 18818675 10.1038/jid.2008.306

[61] Oktarina DAM , van der Wier G , Diercks GFH , Jonkman MF , Pas HH . IgG‐induced clustering of desmogleins 1 and 3 in skin of patients with pemphigus fits with the desmoglein nonassembly depletion hypothesis. Br J Dermatol. 2011;165(3):552‐562. 21692763 10.1111/j.1365-2133.2011.10463.x

[62] Swensson O , Eady RAJ . Morphology of the keratin filament network in palm and sole skin: evidence for site‐dependent features based on stereological analysis. Arch Dermatol Res. 1996;288(2):55‐62. 8932581 10.1007/BF02505044

[63] Vela‐Romera A , Carriel V , Martín‐Piedra MA , et al. Characterization of the human ridged and non‐ridged skin: a comprehensive histological, histochemical and immunohistochemical analysis. Histochem Cell Biol. 2019;151(1):57‐73. 10.1007/s00418-018-1701-x 30099600 PMC6328512

[64] Garzorz‐Stark N , Eyerich K . Molecular diagnostics of hand eczema. Hautarzt. 2019;70(10):760‐765. 31468073 10.1007/s00105-019-4466-9

