# Supplementary material for: Hyperkeratotic hand eczema: Eczema or not?
Source: Contact Dermatitis. 2020 Jun 1;83(3):196–205. doi: 10.1111/cod.13572 (PMC7496397; doi:10.1111/cod.13572)
Supplement: Supplementary file 1 — Supplementary S1 Supporting information. [file COD-83-196-s001.docx]

**Supplement 1**

**Hyperkeratotic hand eczema – eczema or not?**

Klaziena Politiek, Laura Loman, Hendri. H. Pas, Gilles F.H. Diercks, Henny H. Lemmink, Sabrina Z. Jan, Peter C. van den Akker, Maria C. Bolling, Marie L.A. Schuttelaar

| Antigen | Antibody Dilution | | | Supplier |
| --- | --- | --- | --- | --- |
| Keratin 1 | 34bB4 | 1/20 | Novo Castra Laboratories Ltd, Newcastleupon-Tyne, U.K | |
| Keratin 2e | Ks2.398.3.1 | 1/100 | Progen, Heidelberg, Germany | |
| Keratin 5 | CK-5 | 1/200 | Biolegend, San Diego, U.S.A | |
| Keratin 6 | Ks6-KA12 | 1/10 | Progen, Heidelberg, Germany | |
| Keratin 9 | CK9 | 1/10 | Origene, Herford, Germany | |
| Keratin 10 | LHP1 | 1/200 | Santa Cruz biotechnology inc. | |
| Keratin 14 | LL001 | 1/20 | Gift from Dr B*.* Lane*,* Dundee, U.K. | |
| Keratin 15 | LHK15 | 1/100 | Santa Cruz Biotechnology, Dallas, U.S.A | |
| Keratin 16 | LL025 | 1/10 | Gift from Dr B*.* Lane*,* Dundee, U.K. | |
| Keratin 17 | E3 | 1/30 | Novo Castra Laboratories Ltd, Newcastleupon-Tyne, U.K. | |
| DSG1 | 27B2 | 1/20 | Santa Cruz Biotechnology, Dallas, U.S.A | |
| DSG1 | DSG1-P23 | 1/10 | Progen, Heidelberg, Germany | |
| DSG-3 | G194 | 1/40 | Progen, Heidelberg, Germany | |
| Plakophilin-1 | PP1-5C2 | 1/2 | MyBioSource, San Diego, U.S.A | |
| Plakoglobin | 15F11 | 1/1000 | Sigma-Aldrich, Saint-Louis, U.S.A | |
| Desmoplakin (rod) | DP2.17 | 1/200 | MyBioSource, San Diego, U.S.A | |
| Filaggrin | 15C10 | 1/50 | Monosan, Uden, Netherlands | |
| Loricrin | Loricrin | 1/1200 | Biolegend, San Diego, U.S.A | |
| Involucrin | SY5 | 1/100 | Sigma-Aldrich, Saint-Louis, U.S.A | |
| Corneodesmosin | P-20 | 1/50 | Santa Cruz Biotechnology, Dallas, U.S.A | |

**Table S1. Antibodies against keratins, desmosome and cornified cell envelop proteins used in this study**
