# Supplementary material for: Hyperkeratotic hand eczema: Eczema or not?
Source: Contact Dermatitis. 2020 Jun 1;83(3):196–205. doi: 10.1111/cod.13572 (PMC7496397; doi:10.1111/cod.13572)
Supplement: Supplementary file 2 — Supplementary S2 Supporting information. [file COD-83-196-s002.docx]

**Supplement 2**

**Hyperkeratotic hand eczema – eczema or not?**

Klaziena Politiek, Laura Loman, Hendri. H. Pas, Gilles F.H. Diercks, Henny H. Lemmink, Sabrina Z. Jan, Peter C. van den Akker, Maria C. Bolling, Marie L.A. Schuttelaar

**The following 135 candidate genes were analyzed with next-generation sequencing:**

*AAAS, AAGAB, ABCA12, ABHD5, ACD, ADAM10, ALDH3A2, ALOX12B, ALOXE3, AP1S1, AQP5, ATP2A2, BRAF, C16orf57, CAPN12, CARD14, CASP14, CAST, CDSN, CERS3, CHST8, COG6, COL14A1, COL17A1, CSTA, CTC1, CTSC, CYP4F22, DKC1, DSC1, DSC2, DSC3, DSG1, DSG3, DSP, DST, EBP, EDA1, EDAR, EDARADD, ENPP1, ERCC2, ERCC3, ERCC4, ERCC5, ERCC6, ERCC8, EXPH5, FAM83G, FERMT1, FLG, FLG2, GJA1, GJB2, GJB3, GJB4, GJB6, GTF2E2, GTF2H5, HPGD, HRAS, IL36RN, ITGA6, ITGB4, JUP, KANK2, KDSR, KLHL24, KRAS, KRT1, KRT2, KRT5, KRT6A, KRT6B, KRT6C, KRT9, KRT10, KRT14, KRT16, KRT17, KRT83, LAMA3, LAMB3, LAMC2, LIPN, LOR, MAP2K1, MAP2K2, MBTPS2, MPLKIP, NEMO, NIPAL4, NLRP1, NOLA2, NOLA3, NSDHL, PARN, PERP, PIGO, PKP1, PLEC, PNPLA1, POMP, PVRL1, RHBDF2, RNF113A, RSPO1, RTEL1, SASH1, SDR9C7, SERPINB7, SERPINB8, SLC27A4, SLCO2A1, SLURP1, SLURP2, SMARCAD1, SNAP29, SPINK5, SRD5A3, ST14, STS, SULT2B1, TAT, TERC, TERT, TGM1, TGM5, TINF2, TP63, TRPV3, TUFT1, VPS33B, WNT10A, WRAP53.*
