# Supplementary material for: Hyperkeratotic hand eczema: Eczema or not?
Source: Contact Dermatitis. 2020 Jun 1;83(3):196–205. doi: 10.1111/cod.13572 (PMC7496397; doi:10.1111/cod.13572)
Supplement: Supplementary file 3 — Supplementary S3 Supporting information. [file COD-83-196-s003.docx]

**Supplement 3**

**Hyperkeratotic hand eczema – eczema or not?**

Klaziena Politiek, Laura Loman, Hendri. H. Pas, Gilles F.H. Diercks, Henny H. Lemmink, Sabrina Z. Jan, Peter C. van den Akker, Maria C. Bolling, Marie L.A. Schuttelaar

| Patient | Heterozygous gene variants Classification | | Interpretation |
| --- | --- | --- | --- |
| HHE-01 | c.35delG p.(Gly12Valfs*2)  *GJB2* (NM_004004.5)  c.2303delA p.(Lys768Serfs*66)  *EXPH5* (NM_015065.2) | P  P | Autosomal recessive focal palmoplantar keratoderma with severe hearing impairment (OMIM #615028) ^A^  ~0,95% % in European population  Autosomal recessive nonspecific Epidermolysis Bullosa (OMIM #615028)  ^A^ Not present |
| HHE-02 | c.1709T>C p.(Val570Ala)  *ALOX12B* (NM_001139.2) | VOUS | Autosomal recessive congenital ichthyosis with PPK (OMIM #242100)  ^A^ Not present |
| HHE-03 | c.2257G>A, p.(Val753Ile)  *COL14A1* (NM_021110.3) | VOUS | Autosomal recessive punctate PPK, type IB^1^  ^A^ Not present |
| HHE-04 | c.1946A>G p.(His649Arg)  *ERCC6*  (NM_001346440.1) | VOUS | Autosomal recessive Cockayne Syndrome Type B (OMIM#133540)  ^A^ Very low (only 1 individual) ~0,0009% |
| HHE-05 | c.2413G>A p.(Gly805Arg)  *ERCC5* (NM_000123.3) | P | Autosomal recessive xeroderma pigmentosum complementation group G (XPG) and Cockayne syndrome^2^  ^A^  Very low (only 3 individuals) ~ 0,0012% |
| HHE-06 | c.989A>T, p.(Asp330Val)  *CYP4F22 (NM_173483.3)* | VOUS | Autosomal recessive congenital ichhyosis (OMIM# 604777)  ^A^ Low (26 individuals) ~0,02% in European population |
| HHE-07 | No (likely) pathogenic or VUS mutations | |  |

**Table S3. Heterozygous Gene variants in patients with hyperkeratotic hand eczema**

AR: autosomal recessive inheritance; AD: autosomal dominant inheritance; Het: heterozygous; P: Pathogenic; PPK : palmoplantar keratoderma; VOUS: variant of unknown significance. Gene variant classification was based upon criteria as described by Fokkema et al.^3^

^A^ Percentage of healthy control individuals carrying this gene variant in the gnomAD database (<http://gnomad.broadinstitute.org/>)

No pathogenic or likely pathogenic mutations were identified in the 135 PPK-related genes that could be associated with the hyperkeratotic hand eczema phenotype.

**References:**

1. Guo BR, Zhang X, Chen G, Zhang JG, Sun LD, Du WD, et al. Exome sequencing identifies a COL14A1 mutation in a large Chinese pedigree with punctate palmoplantar keratoderma. J Med Genet. 2012;49(9):563–8.

2. Schäfer A, Schubert S, Gratchev A, Seebode C, Apel A, Laspe P, et al. Characterization of three XPG-defective patients identifies three missense mutations that impair repair and transcription. J Invest Dermatol. 2013;133(7):1841–9.

3. Fokkema IFAC, Velde KJ, Slofstra MK, Ruivenkamp CAL, Vogel MJ, Pfundt R, et al. Dutch genome diagnostic laboratories accelerated and improved variant interpretation and increased accuracy by sharing data. Hum Mutat. 2019; Available from: http://dx.doi.org/10.1002/humu.23896
